# Quality assessment standards in artificial intelligence diagnostic accuracy systematic reviews: a meta-research study

**DOI:** 10.1038/s41746-021-00544-y

**Published:** 2022-01-27

**Authors:** Shruti Jayakumar, Viknesh Sounderajah, Pasha Normahani, Leanne Harling, Sheraz R. Markar, Hutan Ashrafian, Ara Darzi

**Affiliations:** 1 Department of Surgery and Cancer, Imperial College London, London, UK; 2 Institute of Global Health Innovation, Imperial College London, London, UK; 3 Department of Thoracic Surgery, Guy’s Hospital, London, UK

**Keywords:** Medical research, Diagnosis, Computational science

## Abstract

Artificial intelligence (AI) centred diagnostic systems are increasingly recognised as robust solutions in healthcare delivery pathways. In turn, there has been a concurrent rise in secondary research studies regarding these technologies in order to influence key clinical and policymaking decisions. It is therefore essential that these studies accurately appraise methodological quality and risk of bias within shortlisted trials and reports. In order to assess whether this critical step is performed, we undertook a meta-research study evaluating adherence to the Quality Assessment of Diagnostic Accuracy Studies 2 (QUADAS-2) tool within AI diagnostic accuracy systematic reviews. A literature search was conducted on all studies published from 2000 to December 2020. Of 50 included reviews, 36 performed the quality assessment, of which 27 utilised the QUADAS-2 tool. Bias was reported across all four domains of QUADAS-2. Two hundred forty-three of 423 studies (57.5%) across all systematic reviews utilising QUADAS-2 reported a high or unclear risk of bias in the patient selection domain, 110 (26%) reported a high or unclear risk of bias in the index test domain, 121 (28.6%) in the reference standard domain and 157 (37.1%) in the flow and timing domain. This study demonstrates the incomplete uptake of quality assessment tools in reviews of AI-based diagnostic accuracy studies and highlights inconsistent reporting across all domains of quality assessment. Poor standards of reporting act as barriers to clinical implementation. The creation of an AI-specific extension for quality assessment tools of diagnostic accuracy AI studies may facilitate the safe translation of AI tools into clinical practice.

## Introduction

With ever-expanding applications for the use of artificial intelligence (AI) in healthcare, interest in its capabilities to analyse and interpret diagnostic tests has increased. AI-driven approaches to the interpretation of diagnostic tests have the potential to overcome several current limitations on clinical review availability, time to diagnosis, diagnostic accuracy and consistency. Recently, various deep learning algorithms have demonstrated comparable or superior performance in the analysis of radiological findings as compared to experts^1^. In conjunction with AI, clinical diagnosticians are capable of improving measures of diagnostic accuracy (such as sensitivity and specificity, area under the curve, positive predictive and negative predictive values) as well as minimising inter- and intra-observer variability in interpretation. Similar studies have also been conducted in non-radiological diagnostics, including AI-driven analysis of endoscopic, retinal and histopathological images^2–4^. As studies examining AI-driven approaches to diagnostic interpretation have become prevalent, systematic reviews have increasingly been published to amalgamate and report these results. Given the diversity and heterogeneity of existing AI techniques, with further rapid expansion expected, clinicians and policymakers may find it difficult to interpret these results and implement these models in their clinical practice. Because of the substantial reliance of these models on data, the quality, quantity and type of data are all important in ensuring high algorithmic accuracy. Additionally, it is prudent to ensure included studies are of high methodological quality and employ rigorous standards of outcome reporting, as they may be influential in altering guidelines or prompting significant policy change. On the other hand, poor quality studies with a lack of transparent reporting may lead to scepticism within healthcare professionals and members of the public, therefore, leading to unnecessary delays in technological adoption. It is therefore imperative that authors of systematic reviews critically appraise literature using an evidence-based, validated quality assessment tool to enable adequate comparison between studies. In this context of rapidly evolving research techniques coupled with scientific and technological progress, assessing the use of and adherence to existing quality assessment tools can offer valuable insights into their usefulness and relevance. Furthermore, understanding the limitations of these tools is pertinent to ensuring necessary amendments can be made to best match current scientific needs.

The most widely used guideline for the methodological assessment of systematic reviews and meta-analyses is the QUADAS tool. QUADAS was created in 2003 and revised in 2011 (QUADAS-2) to categorise the fourteen questions in the original tool into four domains covering flow and timing, reference, standard and patient selection. Each domain is evaluated for biases and the first three are also assessed for applicability^5,6^. However, the applicability of QUADAS-2 for AI-specific studies is unknown. These studies differ methodologically from conventional trials and consist of distinctive features, techniques and a different entity of analytical challenges. Given the differences in study design and outcome reporting, the areas of potential bias are also likely to differ substantially. However, despite these assumptions, there have been no formal studies examining the adherence and suitability of QUADAS-2 in this genre of study. Moreover, there has not been a similar evaluation with respect to emerging AI-centred quality appraisal tools, such as the Radiomics Quality Score (RQS), which was specifically designed for studies reporting on algorithm-based extraction of features from medical images^7^.

Meta-research studies have been increasingly undertaken to evaluate the processes of research and the quality of published evidence, which facilitates the advancement of existing scientific standards. For example, Frank et al. evaluated the correlation between and publication characteristics and found that factors given high importance when assessing study reliability, such as journal impact factor, are not necessarily accurate markers of “truth”^8^. Such studies are imperative to highlight areas for improvement within research practices and lead to changes in guidelines, reporting standards and regulations. Moreover, recent literature has also underscored the importance of modifying and adapting current research methodologies in line with the digital shift in healthcare^9^. Thus, assessing the adherence to QUADAS-2 in current systematic reviews on diagnostic accuracy in AI studies is an important process in understanding its limitations and evaluating the present applicability of this tool in a digital era.

Therefore, the primary aim of this meta-research study is to evaluate adherence to the QUADAS-2 tool within systematic reviews of AI-based diagnostic accuracy. The secondary aims include (i) assessing the applicability of QUADAS-2 for AI-based diagnostic accuracy studies, (ii) identifying other tools for methodological quality assessment and (iii) identifying key features that an AI-specific quality assessment tool for diagnostic accuracy reporting should incorporate.

## Results

### Literature search

**Fig. 1:**
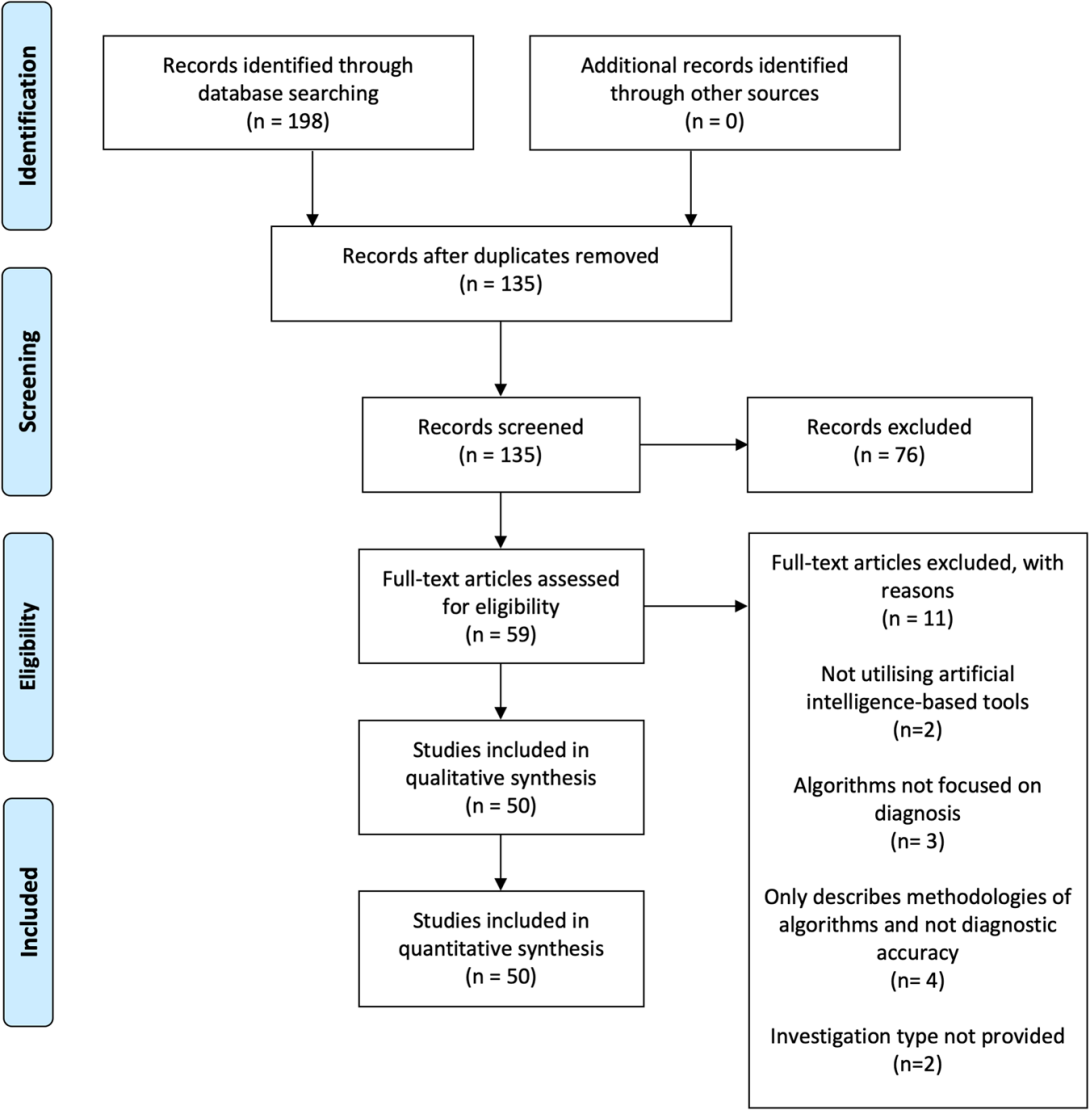
PRISMA flow diagram for systematic literature search and study selection. PRISMA Preferred Reporting Items for Systematic Reviews and Meta-Analyses.

The search yielded 135 papers after the removal of duplicates, of which 48 met the eligibility criteria (Fig. 1). Of 87 excluded, 32 were entirely irrelevant to artificial intelligence, 39 focused on prognostication or prediction, 12 were not systematic reviews and 4 were protocols for systematic reviews. Three papers were excluded upon full-text review as the systematic reviews focussed upon prediction models. Two papers were excluded due to a lack of focus on AI-based diagnostics. Four studies were excluded as they solely discussed the types and methodologies of AI-based tools. Two studies were excluded as they did not specify the investigation type.

### Study characteristics

A total of 1110 studies were included across all 48 systematic reviews, with an average of 23 studies within each systematic review (range: 2–111 studies). The full study characteristics are provided in Tables 1–4. Twenty-three reviews analysed axial imaging, nine analysed non-axial imaging, three analysed digital pathology, two analysed waveform data in the form of electrocardiograms (ECG) and fifteen analysed photographic images. Of these photographic images, six analysed endoscopic images, four analysed skin lesions and five analysed fundus photography or optical coherence tomography.

**Table 1:** Systematic reviews of artificial intelligence-based diagnostic accuracy studies in axial imaging.

Author	Specialty	Included studies	Input variables	Diagnosis
Nayantara 2020^46^	Hepatology	25	CT	Liver lesions
Cho 2020^11^	Oncology	12	MRI	Cerebral metastases
Crombé 2020^47^	Oncology	52	CT, CT-PET, MRI, US	Sarcoma
Kunze 2020^48^	Musculoskeletal	11	MRI	ACL and/or meniscal tears
Groot 2020^13^	Musculoskeletal	14	MRI, X-Rays, US	X-Ray: Fracture detection and/or classification MRI: meniscal/ligament tears, tuberculous vs pyogenic spondylitis US: lateral epicondylitis
Steardo Jr 2020^34^	Psychiatry	22	fMRI	Schizophrenia
Ninatti 2020^49^	Oncology	24	CT, PET-CT	Molecular therapy targets
Ursprung 2020^10^	Oncology	57	CT, MRI	Renal cell carcinoma
Halder 2020^50^	Respiratory Medicine	45	CT	Lung nodules
Li 2019^51^	Respiratory Medicine	26	CT	Lung nodule detection and/or classification
Azer 2019^52^	Hepatology / Oncology	11	CT, MRI, US, Pathology slides	Hepatocellular carcinoma, liver masses
Jo 2019^37^	Neurology	16	MRI, PET, CSF	Alzheimer’s disease
Moon 2019^35^	Psychiatry	43	sMRI, fMRI	Autism spectrum disorder
Sarmento 2020^53^	Neurology	8	CT or MRI	Stroke
Filippis 2019^54^	Psychiatry	35	sMRI, fMRI	Schizophrenia
Langerhuizen 2019^14^	Musculoskeletal	10	CT, X-Rays	Fracture detection and/or classification
Pellegrini 2018^12^	Neurology	111	MRI, CT	Mild cognitive impairment, dementia
Pehrson 2019^55^	Respiratory Medicine	19	CT	Lung nodule
Bruin 2019^36^	Psychiatry	12	sMRI, fMRI	Obsessive-compulsive disorder
McCarthy 2018^56^	Neurology	28	MRI	Frontotemporal dementia
Nguyen 2018^57^	Neurology / Oncology	8	MRI	Differentiate glioblastoma and primary CNS lymphoma
Senders 2018^58^	Neurosurgery	14	CT, MRI, history, age, gender	Intracranial masses, tumours
Smith 2017^59^	Musculoskeletal	18	sMRI, fMRI	Musculoskeletal pain

**Table 2:** Systematic reviews of artificial intelligence-based diagnostic accuracy studies in non-axial imaging.

Author	Specialty	Included studies	Input variables	Diagnosis
Li 2020^60^	Respiratory Medicine	15	Chest X-Ray	Pneumonia
Xu 2020^61^	Oncology / Endocrinology	19	US	Malignant thyroid nodules
Yang 2020^62^	Musculoskeletal	9	X-Rays	Fractures
Groot 2020^13^	Musculoskeletal	14	MRI, X-Rays, US	X-Ray: Fracture detection and/or classification MRI: meniscal/ligament tears, tuberculous vs pyogenic spondylitis US: lateral epicondylitis
Li 2020^63^	Oncology	10	US	Malignant breast masses
Azer 2019^52^	Hepatology / Oncology	11	CT, MRI, US, Pathology slides	Hepatocellular carcinoma, liver masses
Harris 2019^30^	Respiratory Medicine	53	Chest X-Ray	Tuberculosis
Zhao 2019^64^	Endocrinology	5	Ultrasound	Thyroid nodules
Langerhuizen 2019^14^	Musculoskeletal	10	X-Rays, CT	Fracture detection and/or classification

**Table 3:** Systematic reviews of artificial intelligence-based diagnostic accuracy studies in photographic images.

Author	Specialty	Included studies	Input variables	Diagnosis
Bang 2020^65^	Gastroenterology	8	Endoscopic images	H. Pylori infection
Mohan 2020^66^	Gastroenterology	9	Endoscopic images	Gastrointestinal ulcers/haemorrhage
Hassan 2020^67^	Gastroenterology	5	Colonoscopic images	Polyps
Lui 2020^68^	Gastroenterology	18	Colonoscopy images	Polyps
Lui 2020^69^	Gastroenterology	23	Endoscopic images	Neoplastic lesions, Barrett’s oesophagus, squamous oesophagus, H. Pylori status
Wang 2020^70^	Ophthalmology	24	Fundus photography	Diabetic Retinopathy
Soffer 2020^71^	Gastroenterology	10	Wireless Capsule Endoscopic images	Detection of ulcers, polyps, bleeding, angioectasia
Islam 2020^72^	Ophthalmology	31	Fundus photography	Retinal vessel segmentation
Islam 2020^73^	Ophthalmology	23	Fundus photography	Diabetic retinopathy
Murtagh 2020^74^	Ophthalmology	23	OCT, Fundus photography	Glaucoma
Nielsen 2019^75^	Ophthalmology	11	Fundus photography	Diabetic Retinopathy
Marka 2019^38^	Dermatology / Oncology	39	Images of skin lesions	Non-melanoma skin cancer
Ruffano 2018^15^	Dermatology / Oncology	42	Images of skin lesions	Non-melanoma skin cancer
Chuchu 2018^16^	Dermatology / Oncology	2	Images of skin lesions	Melanoma
Rajpara 2009^76^	Dermatology / Oncology	30	Images of skin lesions	Melanoma

**Table 4:** Systematic reviews of artificial intelligence-based diagnostic accuracy studies in pathology images.

Author	Specialty	Included studies	Input variables	Diagnosis
Azam 2020^17^	Pathology	25	Histology samples	Varied—dysplasia, malignancy, challenging diagnoses, identification of small objects, miscellaneous
Mahmood 2020^20^	Oncology / ENT/ Maxfax	11	Histology samples	Malignant head and neck lesions
Azer 2019^52^	Hepatology / Oncology	11	CT, MRI, US, Pathology slides	Hepatocellular carcinoma, liver masses

The most common AI techniques used within the studies comprising the systematic reviews include support vector machines and artificial neural networks, specifically convolutional neural networks.

### Quality assessment

**Fig. 2:**
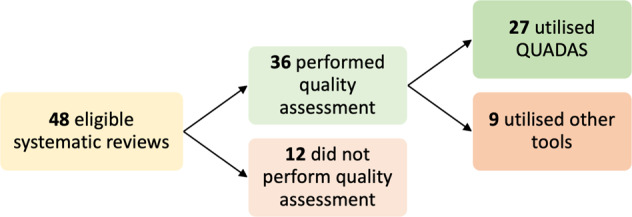
Systematic reviews undertaking quality assessment and utilising QUADAS. QUADAS Quality Assessment of Diagnostic Accuracy Studies.

Thirty-six reviews (75% of studies) undertook a form of quality assessment, of which 27 utilised the QUADAS-2 tool. Further breakdown of quality assessment by study category is detailed below (Fig. 2).

### Diagnostic accuracy of AI in axial imaging

Twenty-three systematic reviews comprising 621 studies reported on the application of AI models to diagnostic axial imaging (Table 1). Of the 23 studies, 14 performed quality assessments with 7 reporting use of the QUADAS tool (Table 5). One study utilised RQS and another study utilised the RQS in addition to QUADAS. Other quality assessment tools used include MINORS (*n* = 3), the Newcastle-Ottawa Score (*n* = 2) and the Jadad Score (*n* = 2).

**Table 5:** Quality āssessment and adherence to QUADAS in systematic reviews of diagnostic accuracy of artificial intelligence in axial imaging.

Study	Modality	Quality assessment	QUADAS	Modifications	Other tools	QUADAS table
Nayantara 2020^46^	CT	No	–	–	–	–
Halder 2020^50^	CT	No	–	–	–	–
Azer 2019^52^	CT, MRI, US, Pathology slides	No	–	–	–	–
Li 2019^60^	CT	No	–	–	–	–
Jo 2019^37^	MRI, PET, CSF	No	–	–	–	–
Sarmento 2019^53^	CT, MRI	No	–	–	–	–
Pehrson 2019^55^	CT	No	–	–	–	–
Bruin 2019^36^	sMRI, fMRI	No	–	–	–	–
Senders 2018^58^	CT, MRI History/age/gender	No	–	–	–	–
Langerhuizen 2019^14^	X-Rays, CT	Yes	No	Yes—modified MINORS	MINORS	–
Smith 2017^59^	sMRI, fMRI	Yes	No	No	Newcastle-Ottawa Scale	–
Crombe 2020^47^	CT, MRI, US	Yes	No	No	Radiomics Quality Score	–
Kunze 2020^48^	MRI	Yes	No	No	MINORS	–
Groot 2020^13^	MRI, X-Rays, US	Yes	No	Yes—modified MINORS	MINORS, TRIPOD	–
Steardo Jr 2020^34^	fMRI	Yes	No	No	Jadad	–
Filippis 2019^54^	sMRI, fMRI	Yes	No	No	Jadad	–
Ninatti 2020^49^	CT, PET-CT	Yes	Yes	No	TRIPOD	Yes
Cho 2020^11^	MRI	Yes	Yes	Yes—modified QUADAS using CLAIM	CLAIM checklist for AI	Yes
McCarthy 2018^56^	MRI	Yes	Yes	No	No	Yes
Moon 2019^35^	sMRI, fMRI	Yes	Yes	No	No	Yes
Pellegrini 2018^12^	MRI, CT	Yes	Yes	Yes—only used QUADAS criteria authors deemed applicable	No	Yes
Nguyen 2018^57^	MRI	Yes	Yes	No	No	Yes (only for bias)
Ursprung 2019^10^	CT, MRI	Yes	Yes	No	Radiomics Quality Score	Yes (multiple raters; no consensus)

Out of the seven studies employing QUADAS, five studies completely reported risk of bias and applicability as per the QUADAS guidelines while one study only reported on the risk of bias. One study provided QUADAS ratings given by each of the study authors, but did not provide a consensus table^10^.

Four studies modified the existing quality assessment tools to improve the suitability and applicability of the tool. Cho et al. tailored the QUADAS tool by applying select signalling questions from CLAIM (Checklist for Artificial Intelligence in Medical Imaging)^11^. Pellegrini and colleagues reported difficulties in finding a suitable quality assessment tool for machine learning diagnostic accuracy reviews and selectively applied items in the QUADAS tool to widen study inclusion^12^. One study modified the MINORS checklist while another study used a modified version of the MINORS checklist in addition to TRIPOD^13,14^.

**Fig. 3:**
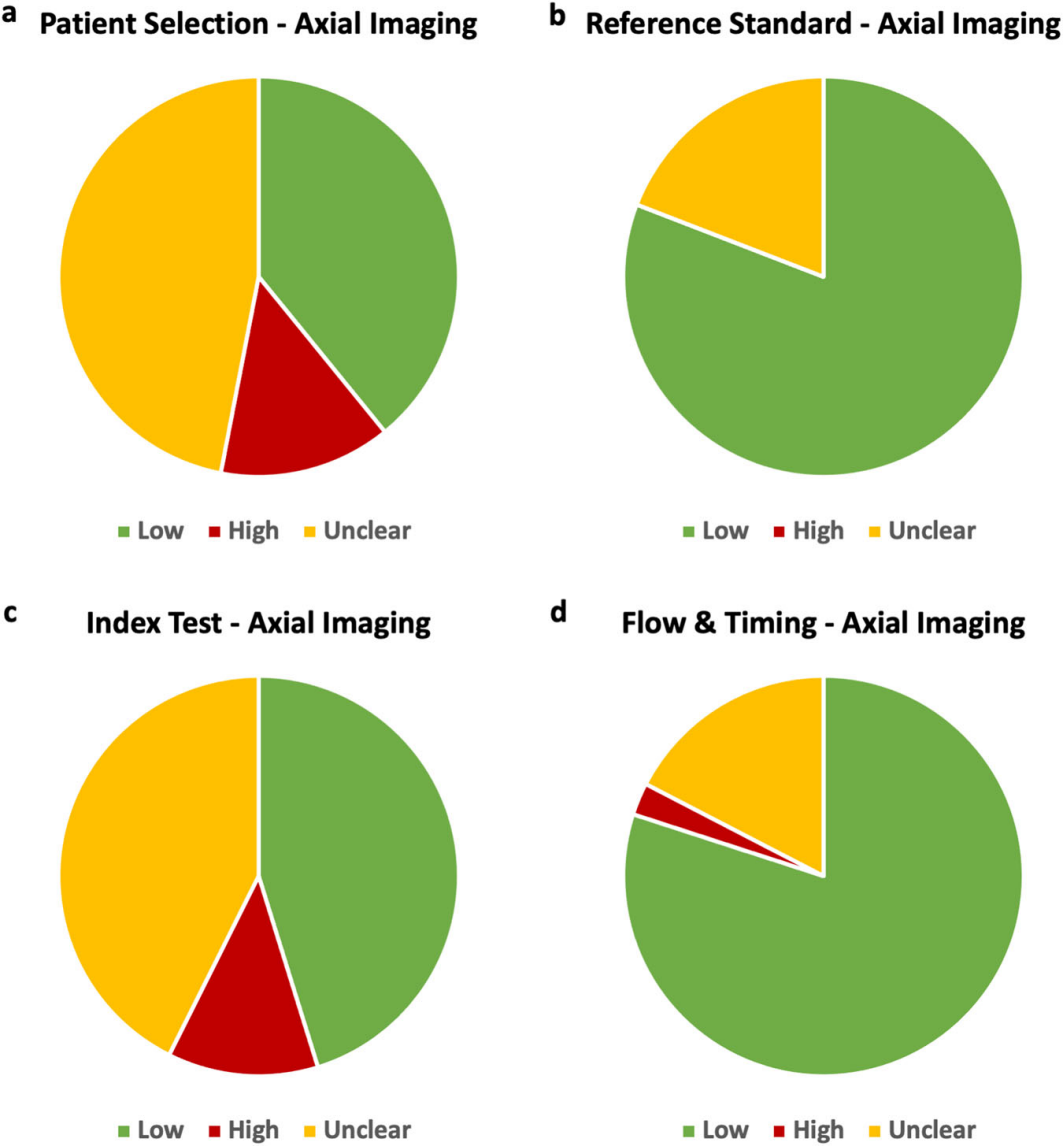
Pie charts demonstrating the risk of bias among axial imaging studies, as assessed through QUADAS. Low, high and unclear risks are shown for the four QUADAS categories: patient selection, reference standard, index test and flow and timing (panels **a, b, c** and **d**, respectively).

Among the 115 studies across six systematic reviews, the patient selection was deemed to pose the highest or most unclear risk of bias. Fifty-four of 115 studies (47%) were considered to have an unclear risk and 16 studies (14%) were classified as high risk of bias (Fig. 3). A high proportion of studies were also considered to pose an unclear risk in the index test domain. Eighty-one percent of studies had a low risk of bias in the reference standard domain with the remainder representing an unclear risk. Concern regarding applicability was generally low for most studies across all five reviews with 78.5%, 87.9% and 93.5% of studies having low concerns of applicability in the patient selection, index test and reference standard domains, respectively.

### Diagnostic accuracy of AI in non-axial imaging

Nine systematic reviews comprising 146 studies reported on the application of AI models to non-axial imaging comprising X-Rays or Ultrasounds (Table 2). Three reviews additionally included studies that also reported on axial imaging.

Of the nine systematic reviews, seven performed quality assessments with five utilising QUADAS (Table 6). The remaining two studies utilised modified versions of the MINORS tools, with one of the studies also utilising TRIPOD as reported under axial imaging.

**Table 6:** Quality assessment and adherence to QUADAS in systematic reviews of diagnostic accuracy of artificial intelligence in non-axial imaging.

Study	Modality	Quality assessment	QUADAS	Modifications	Other tools	QUADAS table
Li 2020^60^	Chest X-Ray	No	–	–	–	–
Azer 2019^52^	CT, MRI, US, Pathology slides	No	–	–	–	–
Langerhuizen 2019^14^	X-Rays, CT	Yes	No	Yes	Modified MINORS	–
Groot 2020^13^	MRI, X-Rays, US	Yes	No	Yes (modified MINORS)	TRIPOD + modified MINORS	–
Xu 2020^61^	US	Yes	Yes	No	No	Yes
Yang 2020^62^	X-Rays	Yes	Yes	No	No	Yes
Li 2020^60^	US	Yes	Yes	No	No	Yes
Harris 2019^30^	Chest X-Ray	Yes	Yes	No	No	Yes
Zhao 2019^64^	US	Yes	Yes	No	No	Yes

**Fig. 4:**
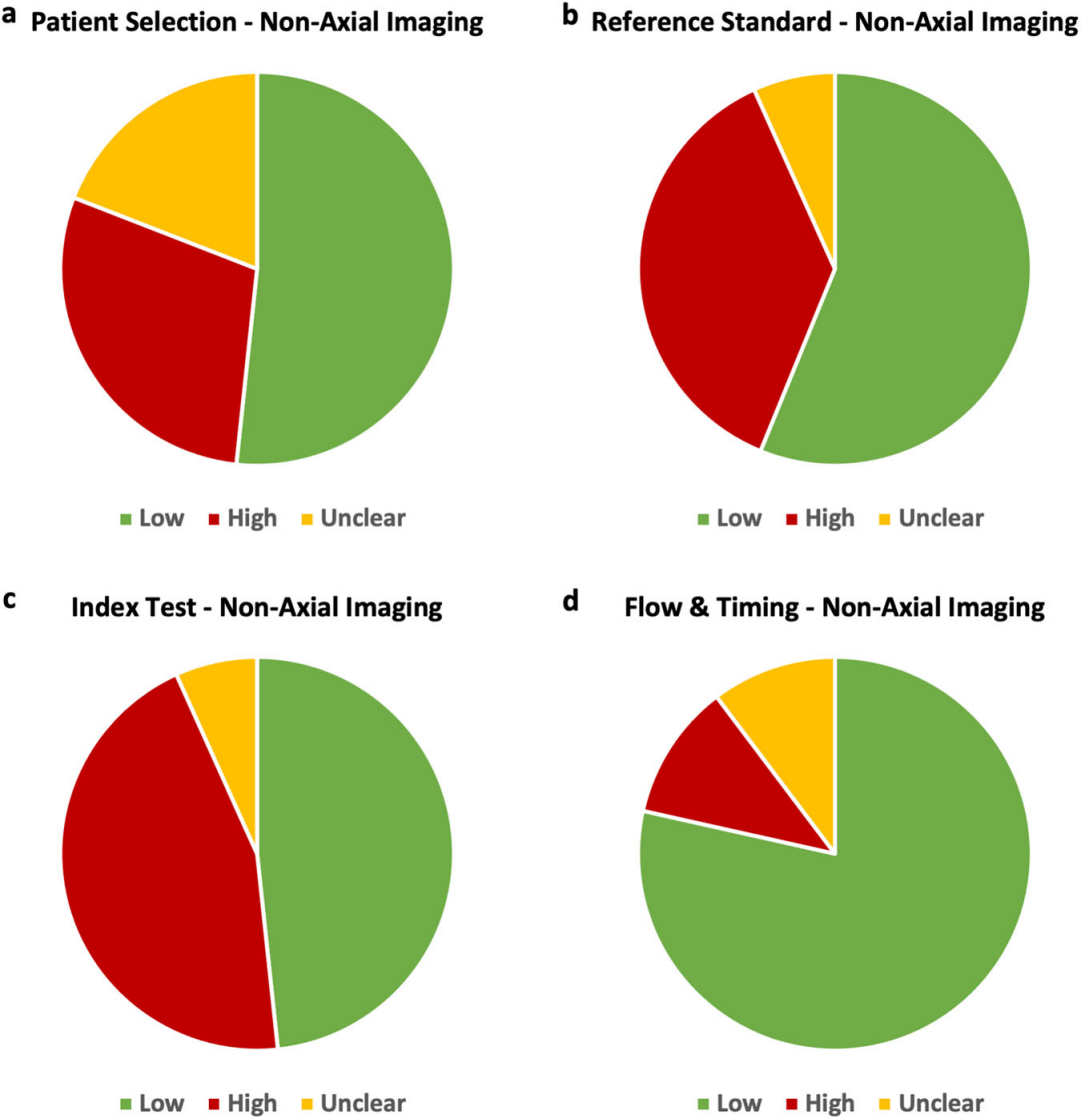
Pie charts demonstrating the risk of bias among non-axial imaging studies, as assessed through QUADAS. Low, high and unclear risks are shown for the four QUADAS categories: patient selection, reference standard, index test and flow and timing (panels **a, b, c** and **d**, respectively).

Among the 89 studies across five systematic reviews, the index test domain posed the highest risk of bias while the patient selection domain posed the most unclear risk of bias (Fig. 4). Concern regarding applicability was generally low for most studies across all five reviews with 79.1%, 79.1% and 90.7% of studies having low concerns of applicability in the patient selection, index test and reference standard domains, respectively.

### Diagnostic accuracy of AI in photographic images

Fifteen systematic reviews comprising 316 studies reported on the application of AI to photo-based diagnostics (Table 3). This consisted of images of skin lesions (*n* = 4), endoscopic images (*n* = 6) and fundus photography or optical coherence tomography (*n* = 5).

Of the 15 systematic reviews, 13 performed quality assessments with 11 utilising QUADAS (Table 7). One study did not report any details on QUADAS while another did not report on applicability concerns and only risk of bias. The remaining two studies utilised the Cochrane Risk of Bias Tool and modified version of the Newcastle-Ottawa scale. In addition, Ruffano et al. and Chuchu et al. adapted the QUADAS tool specifically for non-melanoma skin cancer and melanoma respectively with definitions and thresholds specified by consensus for low and high risk for bias^15,16^.

**Table 7:** Quality assessment and adherence to QUADAS in systematic reviews of diagnostic accuracy of artificial intelligence in photographic images.

Study	Modality	Quality assessment	QUADAS	Modifications	Other tools	QUADAS table
Mohan 2020^66^	Endoscopic images	No	–	–	–	–
Rajpara 2009^76^	Images of skin lesions	No	–	–	–	–
Hassan 2020^67^	Real-time computer-aided detection colonoscopy	Yes	No	No	Cochrane Risk Bias Tool	–
Murtagh 2020^74^	OCT/Fundus photography	Yes	No	Yes—modified Newcastle-Ottawa Scale	Newcastle-Ottawa Scale	–
Bang 2020^65^	Endoscopic images	Yes	Yes	No	–	Yes
Lui 2020^68^	Endoscopic images	Yes	Yes	No	–	Yes
Wang 2020^70^	Fundus photography	Yes	Yes	No	–	Yes
Soffer 2020^71^	Wireless capsule endoscopy	Yes	Yes	No	–	Yes - but not for applicability
Islam 2020^72^	Fundus photography	Yes	Yes	No	–	Yes
Lui 2020^69^	Colonoscopy	Yes	Yes	No	–	Yes
Islam 2020^73^	Fundus photography	Yes	Yes	No	–	Yes
Nielsen 2019^75^	Fundus photography	Yes	Yes	No	–	Yes
Marka 2019^38^	Images of skin lesions	Yes	Yes	No	–	Yes
Ruffano 2018^15^	Images of skin lesions	Yes	Yes	Yes—modified for non-melanoma skin cancers	–	Yes
Chuchu 2018^16^	Images of skin lesions	Yes	Yes	Yes—modified for melanoma	–	Yes

**Fig. 5:**
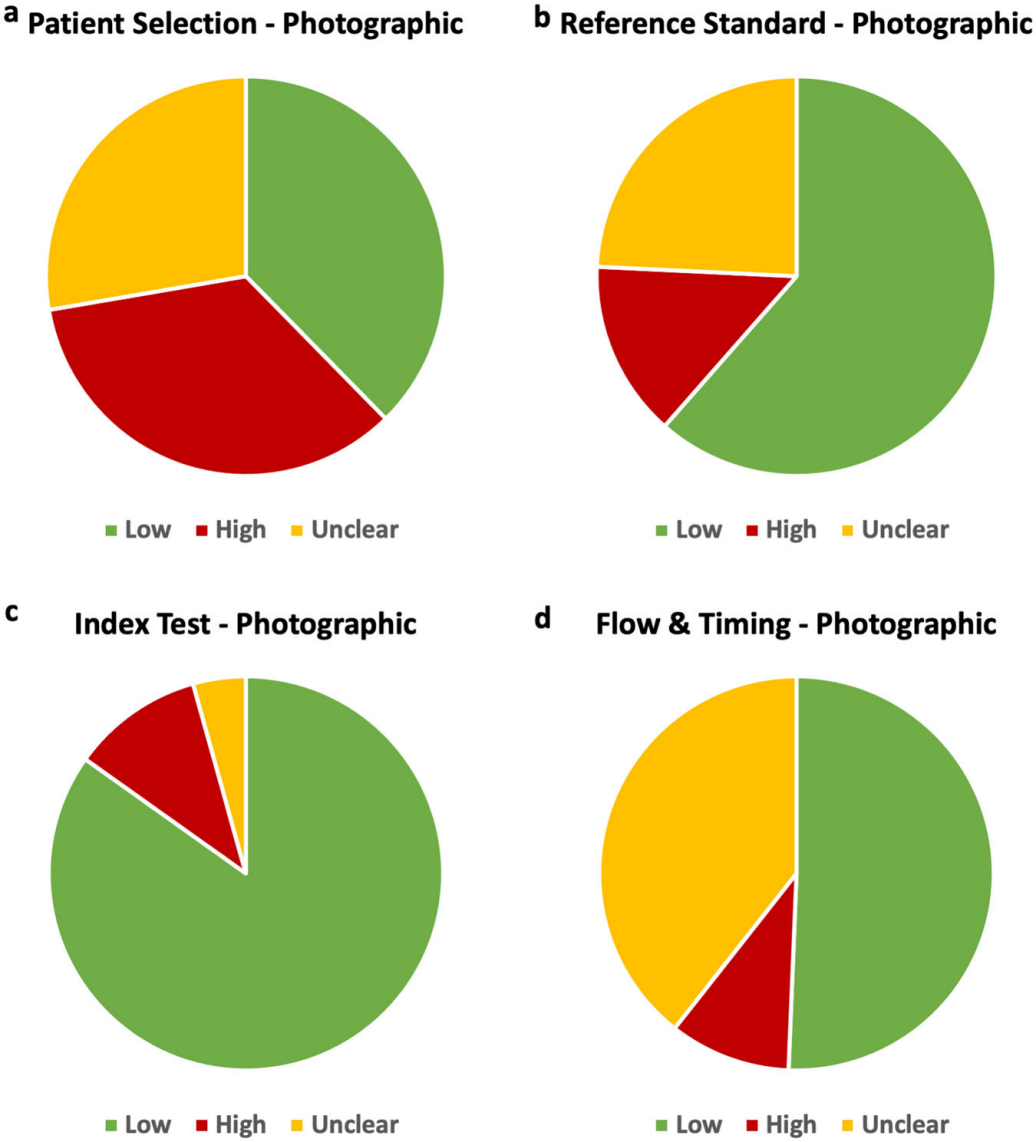
Pie charts demonstrating the risk of bias among photographic images studies, as assessed through QUADAS. Low, high and unclear risks are shown for the four QUADAS categories: patient selection, reference standard, index test and flow and timing (panels **a, b, c** and **d**, respectively).

Among the 231 studies across 11 systematic reviews, the patient selection domain contained the highest risk of bias while the flow and timing domain posed the most unclear risk of bias (Fig. 5). Concern regarding applicability was high or unclear in the patient selection domain for the majority of studies with 54.8% of studies reporting high or unclear applicability concerns. Concerns of applicability were lower in the index test and reference standard domain with 67.5% of studies reporting low concerns in the index test domain and 53.8% in the reference standard domain.

### Diagnostic accuracy of AI in pathology

Three systematic reviews comprising 47 studies reported on the application of AI to pathology. One review examined pathology slides in addition to imaging (Table 4).

Two reviews performed quality assessment utilising QUADAS (Table 8). Mahmood et al. used a tailored QUADAS-2 tool. Only one review provided a tabular display of QUADAS assessment in the recommended format^17^ and reported low risk of bias among the majority of included studies across all domains (Patient Selection: 64% of studies low risk; Index Test: 80% low risk; Reference Standard: 92% low risk; Flow and Timing: 84% low risk) and low concerns regarding applicability.

**Table 8:** Quality āssessment and adherence to QUADAS in systematic reviews of diagnostic accuracy of artificial intelligence in pathology.

Study	Modality	Quality assessment	QUADAS	Modifications	Other tools	QUADAS table
Azam 2020^17^	Histology samples	Yes	Yes	No	No	Yes
Mahmood 2020^20^	Histology samples	Yes	Yes	Yes—modified QUADAS	No	No
Azer 2019^52^	Histology samples	No	–	–	–	–

### Diagnostic accuracy of AI in waveform data

Two systematic reviews comprising 44 primary studies reported on AI algorithms to diagnose pathology from ECGs^18,19^ (Table 9). Both utilised QUADAS-2 and adhered to reporting standards. The risk of bias was low across the majority of included studies with no studies classed a high risk of bias in the patient selection or reference standard domain. Two studies in the index test domain and one study in the flow and timing domain were deemed high risk.

**Table 9:** Quality āssessment and adherence to QUADAS in systematic reviews of diagnostic accuracy of artificial intelligence in waveform data.

Study	Modality	Quality assessment	QUADAS	Modifications	Other tools	QUADAS table
Iannattone 2020^18^	Eectrocardiogram	Yes	Yes	No	No	Yes
Sprockel 2018^19^	Electrocardiogram samples	Yes	Yes	No	No	Yes

### Perceived limitations

Thirteen studies reported an inability to provide systematic quality assessment or evaluate certain biases as a limitation in their study (Supplementary Fig. 1). Specifically, these included concerns around size and quality of the dataset, including its real-world clinical applicability; for example including a whole tissue section instead of the portion of interest only^20^ and providing samples from multiple centres across different demographic populations to improve the generalisability of the model. Appropriate separation of a dataset into training, validation and test sets without overlap was also highlighted as an area needing evaluation, as an overlap between datasets would lead to higher accuracy rates. Eight reviews modified or tailored pre-existing quality assessment tools to customise it to the methodologies and types of studies as reported above.

## Discussion

This study demonstrates that rigorous quality assessment and evaluation of the risk of bias is not consistently carried out in secondary research of AI-based diagnostic accuracy studies. Although considered an essential requirement in secondary research, only 75% of reviews completed quality appraisal, with 56% of papers utilising QUADAS. Although it remains the predominant quality assessment method in this field, the varied use of both new and modified tools (e.g. RQS tool) suggests that the current instruments may not address all the quality appraisal considerations for AI-centred diagnostic accuracy studies. While the primary aim of this paper was to determine adherence to QUADAS guidelines, we also sought to gain a deeper understanding of the reasons behind low adherence to QUADAS in its current form in AI studies. To achieve clinical utility and generalisability, these studies must include data that bears resemblance to the interplay of numerous phenotypical differences contributing to the outcome and adequately reflects the population.

**Fig. 6:**
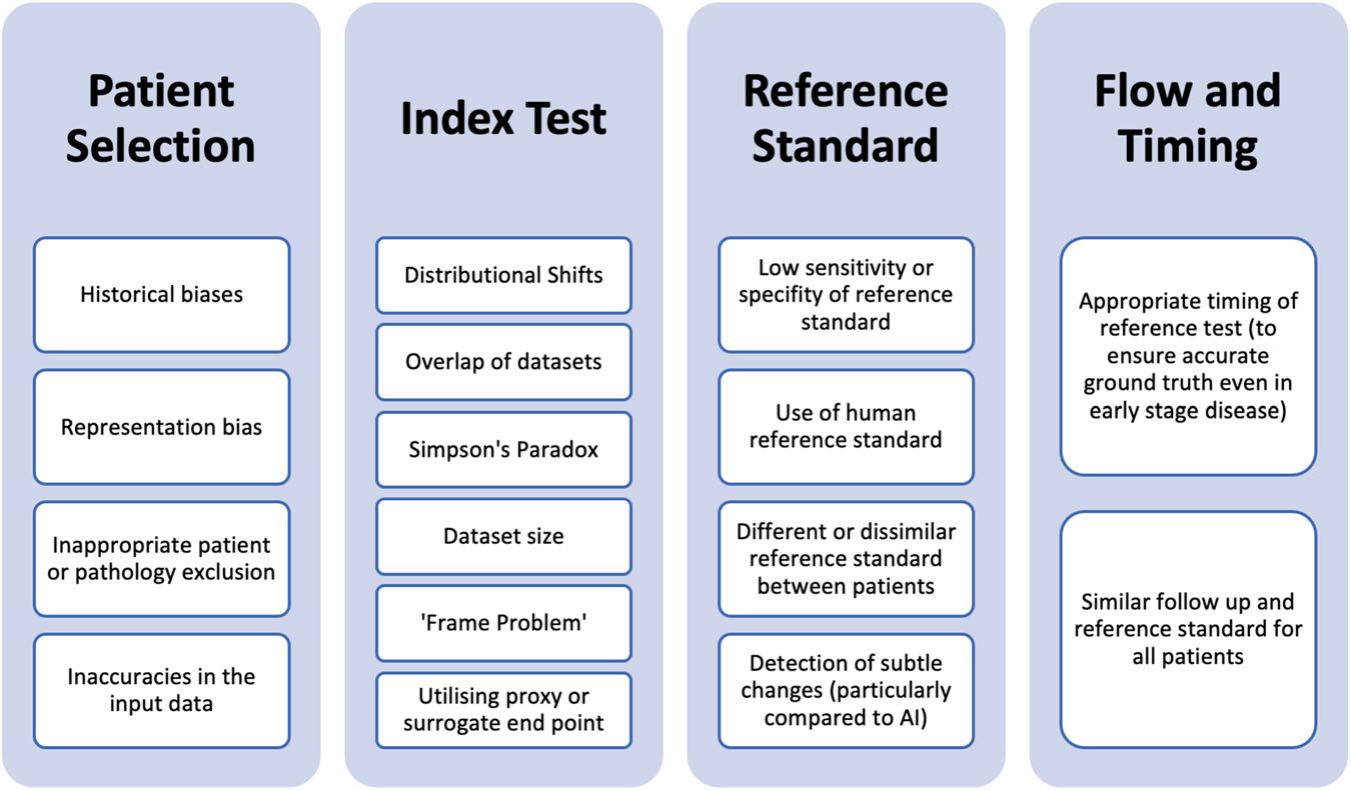
Types of biases affecting quality and applicability of artificial intelligence-based diagnostic accuracy studies. Biases are listed under the QUADAS domain they primarily affect.

In the patient selection domain, 113 studies (26.7% of studies) were deemed high risk and an additional 30.7% of studies were deemed to be of unclear risk of bias. This risk was greatest in studies reporting on photographic images, where 35% of studies were at high risk of bias (Table 10). Factors leading to a high risk of bias in patient selection include poor patient sampling technique and inappropriate exclusion of data on a patient or feature level. As AI algorithms rely on previously seen data to identify patterns and generate results, inaccuracies and biases in input data can be perpetuated and augmented by the model and under-representation of certain factors or demographics may result in inferior algorithm performance^21^. Inappropriate representation of patient demographics or socioeconomic factors may also manifest in the algorithm output as discriminate results. This type of bias may be aggravated in photographic images where utilising data from a specific demographic may create blind spots in the AI algorithm, thus amplifying racial biases^22^. For example, employing an AI model to detect dermatological abnormalities on dark skin resulted in higher rates of missed diagnoses further increasing the disparity in diagnosis^23,24^. In addition to a lack of diversity within the input data, there are several other sources of AI-specific biases including historical bias, representation bias, evaluation bias, aggregation bias, population bias and sampling bias which are discussed in detail by Mehrabi et al. and Simpson’s Paradox (Fig. 6)^25^. Although these biases can be present in research employing traditional statistical methodologies, they may be exaggerated in AI-based tools due to the reliance on existing data. Additionally, there are factors contributing to heterogeneity in images including those related to manufacturer-based specifics for image capture, recording, presentation and the reading platform. These biases and sources of heterogeneity in AI research are also highlighted within some of the included systematic reviews as limitations to the adequate analysis.

**Table 10:** Summary of risk of bias across the QUADAS domains.

	Patient selection	Index test	Reference standard	Flow and timing
Axial imaging	14% high risk 47% unclear	12% high risk 49% unclear	0% high risk 19% unclear	3% high risk 17% unclear
Non-axial imaging	29% high risk 19% unclear	37% high risk 7% unclear	45% high risk 7% unclear	11% high risk 10% unclear
Photographic images	35% high risk 28% unclear	11% high risk 4% unclear	24% high risk 14% unclear	39% high risk 10% unclear
Pathology	28% high risk 8% unclear	8% high risk 12% unclear	8% high risk 0% unclear	16% high risk 0% unclear
Waveform data	0% high risk 32% unclear	5% high risk 5% unclear	0% high risk 27% unclear	2% high risk 25% unclear

The creation of AI diagnostic models requires high-quality datasets, which emulate real-world clinical scenarios to ensure accurate and generalisable outcomes. Consequently, unjustified patient exclusion or inappropriate feature selection may overestimate the diagnostic accuracy of the AI model and increase bias. Exclusion of conditions with overlapping traits to the diagnosis being studied may also skew the results and produce inaccurately higher diagnostic accuracy rates, leading to low clinical utility. For example, excluding all inflammatory pathologies of the bowel when developing an AI tool for polyp detection reduces the algorithm’s ability to discriminate between benign polyps and more serious pathologies in a real-world setting (where patients attend a clinical review with a myriad of underlying pathologies)^26^. Similarly, excluding blurry or out-of-focus images may lead to falsely elevated diagnostic accuracy and is not reflective of real-world situations, thereby reducing clinical value. Finally, in comparison to conventional index tests which require a description of sampling methods on a patient only, AI models also require the description of sampling input level data^27^; insufficient description of this may have led to considerable studies presenting an unclear risk of bias.

Within the index test domain, both axial and non-axial imaging studies demonstrated a high risk of bias. This domain pertains to the development and validation of the AI algorithm and interpretation of the generated output. First, distributional shifts between the training, validation and testing datasets can result in the algorithm producing incorrect results with confidence. These shifts can also lead to inaccurate conclusions about the precision of the algorithm if the algorithm is tested inappropriately on a patient cohort for which it was not trained^28^. Second, overlapping datasets can overestimate diagnostic accuracy in comparison to using external validation data. Third, given the heterogenous nature of large datasets necessary for AI, there is an increased possibility of confounding factors amongst the data. If the model does not appropriately address causal relations between different factors, this can lead to Simpson’s Paradox, which arises when inferences are made from aggregated analysis of heterogenous data comprised of multiple subgroups onto individual subgroups. Separating the dataset into different groups based on confounding variables provides a different result compared to analysing all the data together^25^. Finally, the size of the dataset is particularly important for AI models as smaller datasets may provide lower diagnostic accuracy and result in poor generalisability^29^. Additionally, if the AI is not trained on all the varied presentations of a condition, straightforward diagnoses may not be detected by the algorithm, a flaw also known as the ‘Frame Problem’. Specific signalling questions addressing these potential areas of concern may be useful in identifying and characterising potential sources of bias and determining model generalisability.

Forty-eight studies (11.4%) posed a high risk of bias in the reference standard domain. Though non-axial imaging studies appeared to be disproportionately at higher risk of bias in this domain, all studies resulted from one systematic review^30^. Although overall low risk, this domain contains several potential sources of bias for AI-specific studies of diagnostic accuracy. Determination of an appropriate reference standard or ‘ground truth’ for training models requires consideration of the best available evidence and may involve amalgamating clinical, radiological and laboratory data^29^. Comparison of AI against a human reference standard may be utilised, although should be avoided as a sole reference standard if an alternative test providing higher sensitivity and specificity is feasible. For example, 32 of 33 studies in Harris et al. were at high risk of bias due to the reference standard comprising human interpretation of the chest X-ray without the use of sputum culture confirmation^30^. When utilising a human reference standard, the number and experience of operators and presence of interobserver variability should be clearly detailed. Ideally, the reference standard should include multiple annotations from different experts to reduce subjectivity and account for interobserver variability^20^. This is particularly important in the context of AI given its potential capabilities in detecting disease more accurately than human operators and identifying subtle changes or patterns not detectable by human operators^1,31–33^. In the case of models pertaining to early disease detection, a reference standard comprising a combination of investigations including repeat tests at varying time points may be required.

Finally, the domain covering flow and timing evaluated the time between the reference standard and index test, parity of reference standard assessment amongst all participants and inappropriate exclusion of study patients from the final study results. Within this domain, studies performed reasonably well with only 37 studies (8.8%) recorded as high risk of bias. However, methodologies relating to study flow and standards of timing vary in AI-based studies representing a different risk of bias. For example, neuropsychiatric studies utilising AI have been able to detect the presence of early cognitive changes or aid the diagnosis of psychiatric disorders through identification of otherwise indiscernible changes in structural or functional neuroimaging^34–36^. In mild or initial stages of the disease, AI may actually be more discriminant than the reference standard in identifying early variations or subtle patterns^34,37^. Therefore, the timing of the reference standard in relation to the index test is imperative and may need to be scheduled at a later date to ensure the diagnosis reflected by the reference standard is accurate. Furthermore, variation in reference standards used in positive cases compared to negative cases may cause issues when determining the diagnostic accuracy of AI models. For example, histopathology results may be used to diagnose malignancy but performing a biopsy on obviously non-cancerous lesions presents ethical concerns; and as a result, less invasive but potentially less accurate confirmatory reference standard tests are utilised instead^38^. However, using reference standards that significantly vary in accuracy, such as clinical follow-up only in contrast to tissue diagnosis may cause verification bias i.e. false negatives may actually be classed as true negatives and inflate estimates of accuracy. In these cases where an alternative reference standard is required, utilising an investigation with high negative predictive value such as clinical follow-up with a PET scan to rule out malignancy may be suitable^39^. However, in AI-based studies, additional considerations have to be given for similarities between the ground truth used to train the model and the reference standard used to validate and test the model. If there are considerable disparities between the two, the model may be erroneously deemed inadequate.

Perceived limitations of current quality assessment tools highlight the need for an AI-specific guideline to evaluate diagnostic accuracy studies. Algorithm and input data quality, real-world clinical applicability and algorithm generalisability are important sources of bias that need to be addressed in an adapted AI-specific tool. Quality assessment tools similar to QUADAS are currently being modified to match the evolving landscape of research. For example, STARD (Standards for Reporting of Diagnostic Accuracy Studies), is currently being extended to develop the STARD-AI guidelines to specifically appraise AI-based diagnostic accuracy studies^27^. Additionally, AI extensions to TRIPOD (Transparent Reporting of a Multivariable Prediction Model for Individual Prognosis or Diagnosis) and CONSORT (Consolidated Standards of Reporting Trials) have been published, and SPIRIT-AI (Standard Protocol Items: Recommendations for Interventional Trials) is in progress^40–42^. While our main message is demonstrating a lack of adherence to QUADAS, the heterogeneity seen amongst the studies highlights the confusion on how to best report studies of diagnostic accuracy in AI. This suggests a need to generate a new checklist, which can accommodate AI-specific needs and the changing paradigm of research in a digitally driven world.

This review demonstrates the incomplete uptake of quality assessment tools in AI-centred diagnostic accuracy reviews and highlights variations in AI-specific methodological aspects and reporting across all domains of QUADAS in particular. These factors include generalisability and diversity in patient selection, development of training, validation and testing datasets, as well as definition and evaluation of an appropriate reference standard. When evaluating study quality, potential biases and applicability of AI diagnostic accuracy studies, it is imperative that systematic reviews consider these factors. Whilst the QUADAS-2 tool explicitly recognises the difficulty in developing a tool generalisable to all studies across all specialties and topics and proposes the author modifies the signalling questions as needed, it is essential to further define these questions for AI studies given complexities in methodology. Given the complexities of implementing such tools in practice, it is imperative to have robust tools to evaluate these AI tools to ensure high diagnostic value and seamless translation into a clinical setting^43^.

We propose the creation of a QUADAS-AI extension emulating the successful development of AI extensions to other quality assessment tools^27,40,41^. QUADAS-AI and STARD-AI may be employed in parallel to harmonise the evaluation of diagnostic accuracy studies. The adoption of a robust and accepted instrument to assess the quality of primary diagnostic accuracy AI studies for integration within a systematic review can offer an evidence-base to safely translate AI tools into a real-world setting that can empower clinicians, industry, policymakers and patients to maximise the benefits of AI for the future of medical diagnostics and care.

## Methods

### Search strategy

An electronic search was conducted for studies in accordance with the Preferred Reporting Items for Systematic reviews and Meta-Analyses (PRISMA) guidelines to identify systematic reviews reporting on diagnostic accuracy studies in AI studies (Fig. 1)^44^. MEDLINE and Embase were systematically searched from January 2000 to December 2020. The search strategy was developed through discussion with experts in healthcare AI and research methodology. A mixture of keywords and MeSH terms were used together with appropriate Boolean operators (Supplementary Table 1). Reference lists of included papers were investigated to identify further studies.

### Study selection

Two independent reviewers screened titles and abstracts for initial inclusion. Studies were included if they met the following inclusion criteria: (1) systematic review (2) reporting on AI studies pertaining to diagnostic accuracy. Commentary articles, conference extracts and narrative reviews were excluded. Studies either examining prognostication or reporting on AI/machine learning (ML) to predict the presence of disease were also excluded. Specifically, diagnostic accuracy studies were defined as research evaluating the ability of a tool to evaluate the current presence or absence of a particular pathology, in contrast to prognostication or prediction studies, which forecast an outcome or likelihood of a future diagnosis. Two reviewers (SJ and VS) independently screened titles and abstracts for potential inclusion. All potential abstracts were subjected to full-text review by two independent reviewers. Disagreements were resolved through discussion with a third independent reviewer (HA).

### Data extraction

Data were extracted onto a standardised proforma by two independent reviewers (VS and SJ). Study characteristics extracted were study author, year, institution, country, journal and journal impact factor. Key AI-related extraction items were identified through examination of recently developed AI extensions to existing quality assessment tools. A consensus was reached amongst authors to ascertain vital items for data extraction including use of QUADAS-2 and/or other quality assessment tools, quality assessment tool adherence, risk of bias within individual studies, modifications to pre-existing tools, use of multiple tools to improve applicability to AI-specific studies and any limitations pertaining to quality assessment expressed by study authors.

Studies were classified into five clinical categories based upon the type of sample evaluated and upon the diagnostic task: (a) axial medical imaging, (b) non-axial medical imaging, (c) histopathological digital records (digital pathology) (d) photographic images and (e) physiological signals.

### Quality assessment

The AMSTAR 2 (A MeaSurement Tool to Assess systematic Reviews) was employed to evaluate the quality of included studies (Supplementary Table 2)^45^.

### Reporting summary

Further information on research design is available in the Nature Research Reporting Summary linked to this article.

## Supplementary information

Supplementary Information

Reporting Summary

## Data Availability

The authors declare that all data included in the results of this study are available within the paper and the Supplementary files.
