# Supplementary material for: Quality assessment standards in artificial intelligence diagnostic accuracy systematic reviews: a meta-research study
Source: NPJ Digit Med. 2022 Jan 27;5:11. doi: 10.1038/s41746-021-00544-y (PMC8795185; doi:10.1038/s41746-021-00544-y)

Supplementary Table 1: Search Strategy

A. OVID Medline 2000 to December 1 2020

1. Artificial intelligence/ or machine learning/ or deep learning/ or “neural networks (computer)”/
2. (deep learning or convolutional or cnn or neural network*).mp. [mp=title, abstract, original title of substance word, subject heading word, floating sub-heading word, keyword heading word, organism supplementary concept word, protocol supplementary concept word, rare disease supplementary concept word, unique identifier, synonyms]
3. 1 or 2
4. (systematic review or review).tw
5. meta analys?s.tw
6. 4 or 5
7. exp “sensitivity and specificity”/ or exp roc curve/
8. diagnostic adj2 accuracy.ab,kf,ti.
9. diagnostic adj2 utility.ab,kf,ti.
10. 7 or 8 or 9
11. 3 and 6 and 10

B. EMBASE 2000 to December 1 2020

1. exp artificial intelligence/
2. (deep learning or convolutional or cnn or neural network*).mp. [mp=title, abstract, heading word, drug trade name, original title, device manufacturer, drug manufacturer, device trade name, keyword, floating subheading word, candidate term word]
3. 1 or 2
4. systematic review or review
5. meta analys?s
6. 4 or 5
7. exp “sensitivity and specificity”/
8. exp area under the curve/
9. diagnostic accuracy
10. diagnostic utility
11. 7 or 8 or 9 or 10
12. 3 and 6 and 11

Supplementary Table 2: Assessment of Systematic Review Quality Using the AMSTAR (A MeaSurement Tool to Assess systematic Reviews) Tool

	1) Question and Inclusion	2) Protocol	3) Study Design	4) Comprehensive Search	5) Study Selection	6) Data Extraction	7) Excluded Study Justification	8) Included Studies Details	9) Risk of Bias	10) Funding Sources	11) Statistical Methods	12) Risk of Bias on Meta-Analysis	13) Effect of Risk of Bias in Studies	14) Explanation for Heterogeneity	15) Publication Bias	16) Conflict of Interest Statement
Azam 2020	Yes	Yes	Yes	Yes	Yes	Yes	Yes	Yes	Yes	No	Yes	No	No	Yes	No	Yes
Azer 2019	Yes	No	Yes	Yes	Yes	Yes	Yes	Yes	No	No	N/A	N/A	No	No	N/A	Yes
Bang 2020	Yes	Yes	Yes	Yes	Yes	Yes	Yes	Yes	Yes	No	Yes	Yes	Yes	Yes	Yes	Yes
Bruin 2019	No	No	No	Yes	No	No	Yes	Yes	No	No	N/A	N/A	No	No	N/A	Yes
Cho 2020	Yes	Yes	Yes	Yes	Yes	Yes	Yes	Yes	Yes	No	Yes	Yes	Yes	Yes	No	Yes
Chuchu 2018	Yes	Yes	Yes	Yes	Yes	Yes	Yes	Yes	Yes	No	N/A	N/A	Yes	Yes	N/A	Yes
Crombé 2020	Yes	Yes	Yes	Yes	Yes	Yes	Yes	Yes	Yes	No	Yes	No	Yes	No	No	Yes
Filippis 2019	Yes	Yes	Yes	Yes	Yes	Yes	Yes	Yes	Yes	No	N/A	N/A	Yes	No	N/A	Yes
Groot 2020	Yes	Yes	Yes	Yes	Yes	Yes	Yes	Yes	Yes	No	N/A	N/A	Yes	No	N/A	Yes
Halder 2020	No	No	No	Partial Yes	No	No	No	No	No	No	No	No	No	No	No	Yes
Harris 2019	Yes	Yes	Yes	Yes	Yes	Yes	Yes	Yes	Yes	Yes	N/A	N/A	Yes	Yes	N/A	Yes
Hassan 2020	Yes	Yes	Yes	Yes	Yes	Yes	Yes	Yes	Yes	Yes	Yes	No	Yes	Yes	Yes	Yes
Iannattone 2020	Yes	Yes	Yes	Yes	Yes	Yes	Yes	Yes	Yes	Yes	Yes	No	Yes	Yes	Yes	Yes
Islam 2020 (Retinal vessel segmentation)	Yes	Yes	Yes	Yes	Yes	Yes	Yes	Yes	Yes	Yes	Yes	No	No	No	No	Yes
Islam 2020 (Diabetic retinopathy)	Yes	Yes	Yes	Yes	Yes	Yes	Yes	Yes	Yes	Yes	Yes	No	No	No	No	Yes
Jo 2019	Yes	No	No	No	No	No	Yes	Yes	No	No	N/A	N/A	No	No	N/A	Yes
Kunze 2020	Yes	Yes	Yes	Yes	Yes	Yes	Yes	Yes	Yes	No	N/A	N/A	No	No	N/A	Yes
Langerhuizen 2019	Yes	Yes	Yes	Yes	Yes	Yes	Yes	Yes	Yes	No	N/A	N/A	No	No	N/A	Yes
Li 2019	Yes	No	Yes	Yes	Yes	Yes	No	Yes	No	No	N/A	N/A	No	No	N/A	Yes
Li 2020 (Breast)	Yes	Yes	Yes	Yes	Yes	Yes	Yes	Yes	Yes	No	Yes	Yes	Yes	Yes	Yes	Yes
Li 2020 (Pneumonia)	Yes	Yes	Yes	Yes	Yes	Yes	Yes	Yes	Yes	No	Yes	No	Yes	Yes	Yes	Yes
Lui 2020 (Colorectal)	Yes	Yes	Yes	Yes	Yes	Yes	Yes	Yes	Yes	No	Yes	No	Yes	Yes	Yes	Yes
Lui 2020 (Upper GI)	Yes	Yes	Yes	Yes	Yes	Yes	Yes	Yes	Yes	No	Yes	No	Yes	Yes	Yes	Yes

	1) Question and Inclusion	2) Protocol	3) Study Design	4) Comprehensive Search	5) Study Selection	6) Data Extraction	7) Excluded Study Justification	8) Included Studies Details	9) Risk of Bias	10) Funding Sources	11) Statistical Methods	12) Risk of Bias on Meta-Analysis	13) Effect of Risk of Bias in Studies	14) Explanation for Heterogeneity	15) Publication Bias	16) Conflict of Interest Statement
Mahmood 2020	Yes	Yes	Yes	Yes	Yes	Yes	Yes	Yes	Yes	No	N/A	N/A	Yes	No	N/A	Yes
Marka 2019	Yes	Yes	Yes	Yes	Yes	Yes	Yes	Yes	Yes	No	N/A	N/A	Yes	No	N/A	Yes
McCarthy 2018	Yes	Yes	Yes	Yes	Yes	Yes	Yes	Yes	Yes	No	N/A	N/A	Yes	Yes	N/A	Yes
Mohan 2020	Yes	Yes	Yes	Yes	Yes	Yes	Yes	Yes	Yes	No	Yes	No	Yes	Yes	No	Yes
Moon 2019	Yes	Yes	Yes	Yes	Yes	Yes	Yes	Yes	Yes	No	Yes	No	Yes	Yes	No	Yes
Murtagh 2020	Yes	Yes	Yes	Yes	Yes	Yes	Yes	Yes	Yes	No	Yes	Yes	Yes	Yes	Yes	Yes
Nayantara 2020	No	No	No	Yes	No	No	Yes	Yes	No	No	N/A	N/A	No	No	N/A	Yes
Nguyen 2018	Yes	Yes	Yes	Yes	Yes	Yes	Yes	Yes	Yes	No	Yes	No	Yes	No	No	Yes
Nielsen 2019	Yes	Yes	Yes	Yes	Yes	Yes	Yes	Yes	Yes	No	N/A	N/A	Yes	No	N/A	Yes
Ninatti 2020	Yes	Yes	Yes	Yes	Yes	Yes	Yes	Yes	Yes	No	N/A	N/A	Yes	No	N/A	Yes
Pehrson 2019	Yes	No	Yes	Yes	Yes	Yes	No	Yes	No	No	N/A	N/A	No	No	N/A	Yes
Pellegrini 2018	Yes	Yes	Yes	Yes	Yes	Yes	No	Yes	No	No	Yes	No	No	No	No	Yes
Rajpara 2009	Yes	Yes	Yes	Yes	Yes	Yes	Yes	Yes	Yes	No	N/A	N/A	Yes	Yes	N/A	Yes
Ruffano 2018	Yes	Yes	Yes	Yes	Yes	Yes	Yes	Yes	Yes	No	N/A	N/A	Yes	Yes	N/A	Yes
Sarmento 2019	No	No	No	Yes	No	No	No	No	No	No	N/A	N/A	No	No	N/A	Yes
Senders 2018	Yes	No	Yes	Yes	Yes	Yes	Yes	Yes	No	No	N/A	N/A	No	No	N/A	Yes
Smith 2017	Yes	Yes	Yes	Yes	Yes	Yes	Yes	Yes	Yes	No	N/A	N/A	Yes	No	N/A	Yes
Soffer 2020	Yes	Yes	Yes	Yes	Yes	Yes	Yes	Yes	Yes	No	Yes	No	Yes	Yes	No	Yes
Sprockel 2018	Yes	Yes	Yes	Yes	Yes	Yes	Yes	Yes	Yes	No	N/A	N/A	Yes	Yes	N/A	Yes
Steardo 2020	Yes	Yes	Yes	Yes	Yes	Yes	Yes	Yes	Yes	No	N/A	N/A	Yes	Yes	N/A	Yes
Tan 2020	Yes	Yes	Yes	Yes	Yes	Yes	Yes	Yes	Yes	No	Yes	No	Yes	Yes	No	Yes
Ursprung 2019	Yes	Yes	Yes	Yes	Yes	Yes	Yes	Yes	Yes	No	Yes	No	Yes	Yes	Yes	Yes
Wang 2020	Yes	Yes	Yes	Yes	Yes	Yes	Yes	Yes	Yes	No	Yes	Yes	Yes	Yes	No	Yes
Xu 2020	Yes	Yes	Yes	Yes	Yes	Yes	Yes	Yes	Yes	No	Yes	No	Yes	Yes	Yes	Yes
Yang 2020	Yes	Yes	Yes	Yes	Yes	Yes	Yes	Yes	Yes	No	Yes	No	Yes	Yes	Yes	Yes
Zhao 2019	Yes	Yes	Yes	Yes	Yes	Yes	Yes	Yes	Yes	No	Yes	No	Yes	Yes	Yes	Yes

Supplementary Figure 1: Limitations Perceived by Study Authors on Quality Assessment of Included Studies Grouped by Theme. Thirteen studies in total reported limitations relating to quality assessment. Of these, seven studies stated an overall lack of suitable quality assessment tools for evaluation of artificial intelligence diagnostic accuracy studies as a limitation.

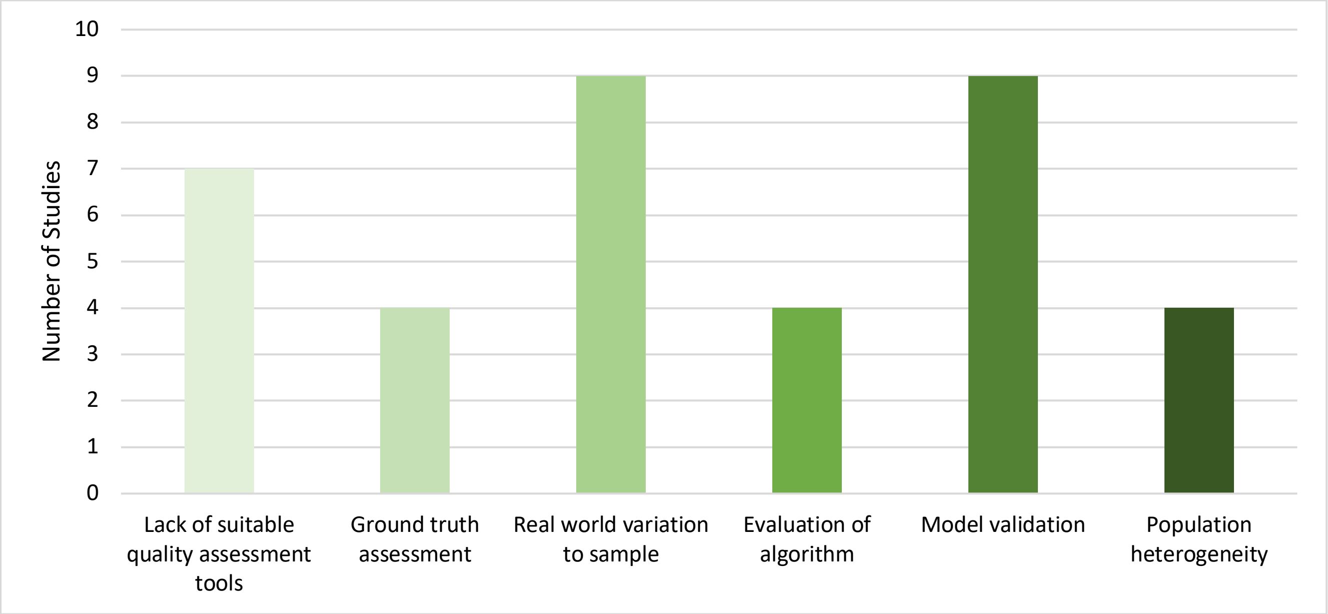

Supplement: Supplementary file 1 — Supplementary Information [file 41746_2021_544_MOESM1_ESM.pdf]
